# Removal of Electrocardiogram Artifacts From Local Field Potentials Recorded by Sensing-Enabled Neurostimulator

**DOI:** 10.3389/fnins.2021.637274

**Published:** 2021-04-12

**Authors:** Yue Chen, Bozhi Ma, Hongwei Hao, Luming Li

**Affiliations:** ^1^National Engineering Laboratory for Neuromodulation, Tsinghua University, Beijing, China; ^2^Precision Medicine & Healthcare Research Center, Tsinghua-Berkeley Shenzhen Institute, Tsinghua University, Shenzhen, China; ^3^International Data Group (IDG)/McGovern Institute for Brain Research at Tsinghua University, Beijing, China; ^4^Institute of Epilepsy, Beijing Institute for Brain Disorders, Beijing, China

**Keywords:** local field potential, ECG, artifact, removal, sensing-enabled neurostimulator

## Abstract

Sensing-enabled neurostimulators are an advanced technology for chronic observation of brain activities, and show great potential for closed-loop neuromodulation and as implantable brain-computer interfaces. However, local field potentials (LFPs) recorded by sensing-enabled neurostimulators can be contaminated by electrocardiogram (ECG) signals due to complex recording conditions and limited common-mode-rejection-ratio (CMRR). In this study, we propose a solution for removing such ECG artifacts from local field potentials (LFPs) recorded by a sensing-enabled neurostimulator. A synchronized monopolar channel was added as an ECG reference, and two pre-existing methods, i.e., template subtraction and adaptive filtering, were then applied. ECG artifacts were successfully removed and the performance of the method was insensitive to residual stimulation artifacts. This approach to removal of ECG artifacts broadens the range of applications of sensing-enabled neurostimulators.

## Introduction

Recently, sensing-enabled neurostimulation has emerged as a technology for long-term observation of brain activities and has paved the way for development of closed-loop neuromodulators and implantable brain-computer interfaces (Stanslaski et al., 2012; Qian et al., 2014; Martini et al., 2020; Ramirez-Zamora et al., 2020). Using sensing-enabled neurostimulators, a seminal series of studies made enormous advances in the mechanisms of deep brain stimulation (DBS) (Trager et al., 2016; Neumann et al., 2017), the effects of closed-loop neuromodulation (Meidahl et al., 2017; Velisar et al., 2019), and the feasibility of implantable brain-computer interfaces (Vansteensel et al., 2016; Golshan et al., 2020). By integrating recordings of LFPs, sensing-enabled neurostimulators have opened a real window into chronic brain activities (Shen, 2014). However, it is difficult to faithfully record LFPs due to electrocardiogram (ECG) and stimulation artifacts (Sorkhabi et al., 2020). Previous studies using sensing-enabled neurostimulators reported considerable data loss due to ECG contamination (Quinn et al., 2015; Trager et al., 2016; Anidi et al., 2018; Hell et al., 2018; Swann et al., 2018). The research of Quinn et al. (2015) showed that six of the sixteen recordings were excluded because of ECG artifacts. In some cases, ECG artifacts could develop in the LFPs recorded during longitudinal follow-ups (Trager et al., 2016). The researchers had to choose those LFP channels free of ECG contamination (Swann et al., 2018), which limited the precision of the targeted recording positions.

ECG artifacts can be attributed to an inadequate common-mode-rejection-ratio (CMRR) of the recording module in a sensing-enabled neurostimulator. For LFP recording, signals were usually differentiated between pairs of contacts on the electrode. The ECG spikes are regarded as common-mode signals that can be rejected by differentiating. To eliminate ECG artifacts, previous studies have shown that the CMRR has to be greater than 60 dB (Sorkhabi et al., 2020). However, achieving a high and stable CMRR is very challenging for implantable devices because power consumption and size of the neurostimulators are very limited. Furthermore, after implantation, a slight leakage of fluid into the neurostimulator can break the symmetry of the differentiated channels, which alters the CMRR (Quinn et al., 2015).

Although there are ample algorithms for removal of ECG artifacts from electrophysiological signals, few can be applied to LFP recordings. Template subtraction calculates the average waveform of ECG artifacts and subtracts it from each spike in contaminated recordings (Zhou et al., 2007; Marker and Maluf, 2014). A previous study used a raw ECG signal and template subtraction to remove the ECG artifacts from LFPs recorded by sensing-enabled neurostimulators (Canessa et al., 2016). Adaptive filtering is another popular method for removing ECG artifacts (Lu et al., 2009; Sweeney et al., 2012). It estimates the noise component and subtracts it from the original recording. Both methods usually require a synchronized ECG waveform for reference. Independent component analysis (ICA) is a widely used blind-source separation method that dispenses with the need for an ECG reference (Mak et al., 2010; Sweeney et al., 2012). However, the recordings of sensing-enabled neurostimulators are usually made without an ECG reference, and the number of LFP channels is too limited (for example, two channels) to perform ICA. Therefore, these methods are unsuitable for removal of ECG artifacts from most LFP recordings. Although there are some blind-source separation methods for single-channel recordings (Sweeney et al., 2012), their robustness is inconclusive due to residual DBS artifacts.

In this study, we propose a method for the removal of ECG artifacts from LFPs. A sensing-enabled neurostimulator was used to record simulated LFPs contaminated with ECG artifacts in saline solution. We modified the recording montages to add a synchronized ECG reference channel. Using the reference channel, we used template subtraction and adaptive filtering to remove ECG artifacts from simulated LFPs. We evaluated the performance of the proposed method of ECG artifact removal and explored the effects of DBS artifacts. The results show that using modified recording montages allows ECG artifacts to be successfully removed, thus revealing the LFP signals.

## Materials and Methods

### Instrumentation Design and Implementation

#### Sensing-Enabled Neurostimulator

**FIGURE 1:**
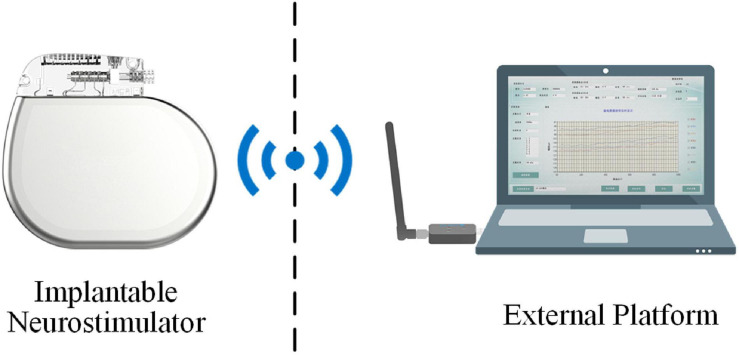
Illustration of the sensing-enabled neurostimulator system.

The LFP recording instrumentation is illustrated in Figure 1. A sensing-enabled neurostimulator (G102RS, Beijing PINS Medical Co., Ltd.) which can be fully implanted for DBS therapy was used. Before sampling, signals were first filtered by a built-in 0.3–250 Hz band-pass filter. Then the signals were sampled by the neurostimulator and wirelessly transmitted to the external PC platform through radio frequency communication. The transmission rate was 250 kbps and the delay was less than 10 ms. The wireless communication distance was approximately 2 m. A rechargeable battery was incorporated into the neurostimulator to ensure long-term recording.

#### Modification of Recording Montages

**FIGURE 2:**
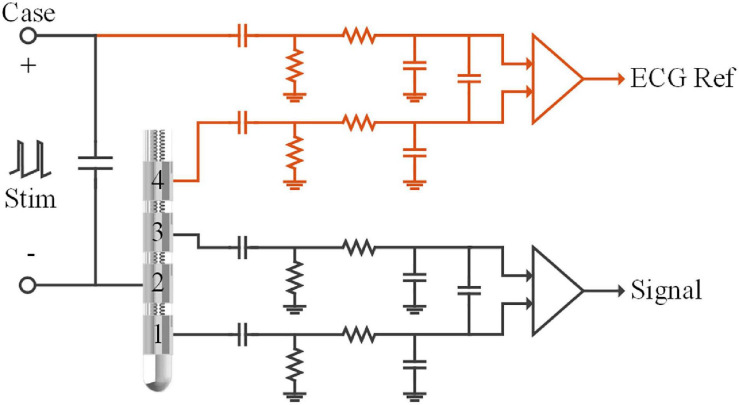
Schematic of the recording module in the sensing-enabled neurostimulator. LFPs were recorded in the bipolar recording channel (between contacts 1 and 3). The ECG signals were recorded in the monopolar recording channel (between the titanium case and contact 4). The capacitors and resistors in each chain formed passive band-pass filters (0.3–250Hz) of the analog-front-end circuits. Monopolar stimulation was delivered between the titanium case and contact 2.

For LFP recording, signals were usually differentiated between pairs of contacts in the electrode, i.e., bipolar recording. For example, in Figure 2, the signal was differentiated between contact 1 and contact 3 in the DBS electrode after the passive filter network. Monopolar stimulation was synchronously delivered between the titanium case of the neurostimulator (anode, implanted in the chest) and the therapeutic contact 2 (cathode). Potentials generated by the stimulation and the ECG source were approximately the same at contacts 1 and 3. Thus, both of the ECG potentials and stimulation pulses could be regarded as common-mode signals in the bipolar recording chain.

To achieve efficient ECG artifact removal, we developed recording montages by adding a channel for the ECG reference in the neurostimulator. As shown in Figure 2 (red lines), a monopolar recording channel was differentiated between the titanium case and another contact in the DBS electrode. In this monopolar channel, ECG signals were synchronously recorded with the LFPs in the bipolar recording channel. Although the monopolar recording montage may yield larger DBS artifacts, recording ECG signals is possible as long as the analog-to-digital converter is not saturated. In the following sections, we describe tests of the feasibility of synchronized bipolar and monopolar recordings during stimulation and evaluate the benefits of the modified recording montages for removal of ECG artifacts.

### Data Recording

#### Simulated Recording Setup

**FIGURE 3:**
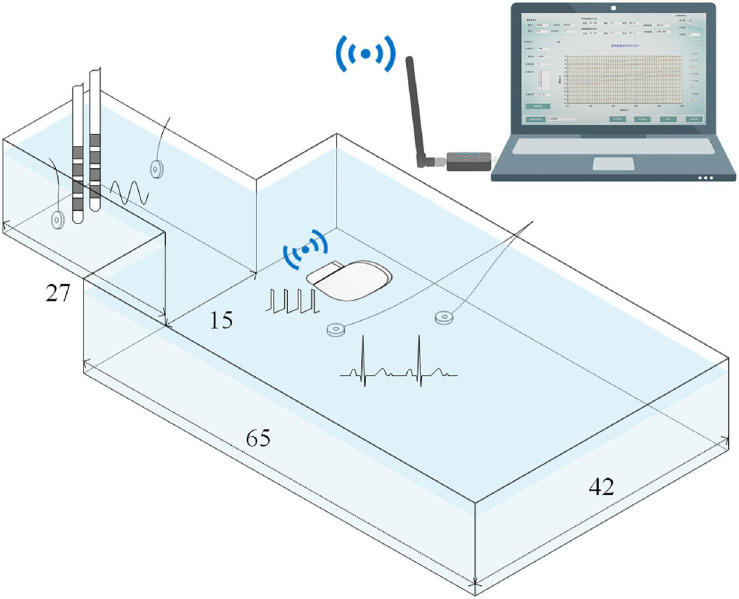
Illustration of the simulated recording setup. The titanium case and the DBS electrodes were fully immersed in the phantom that was filled with saline solution.Phantom size is expressed in centimeters. A 23Hz sinusoidal signal and a standard ECG waveform were delivered by two pairs of Ag/Cl disc-electrodes. The recordings were wirelessly transmitted toa computer.

As shown in Figure 3, to simulate signal recordings after implantation, the neurostimulator’s titanium case and the DBS electrodes were fully immersed in an ASTM phantom (ASTM, 2011) filled with saline solution at room temperature. The DBS electrodes (model L301, Beijing PINS Medical Co., Ltd.) have four platinum-iridium cylindrical contacts. The contacts were 1.3 mm in diameter, 1.5 mm in length, and spaced 0.5 mm apart. Two Ag/Cl disc-electrodes beside the DBS electrodes delivered a 23 Hz sinusoidal signal that simulated an LFP, which was chosen for the following reasons: (1) the beta band (13–35 Hz) of LFPs recorded by sensing-enabled neurostimulators is the most relevant band for revealing the mechanisms of DBS and for developing a closed-loop stimulation strategy (Trager et al., 2016; Meidahl et al., 2017; Neumann et al., 2017; Velisar et al., 2019), and (2) based on previous clinical experience, ECGs mainly contaminate the low-frequency band (below 100 Hz) in these specific LFPs, especially in the beta band. For the relevant signals in the high-frequency band (above 100 Hz), a high-pass filter could remove the ECG artifacts (Redfern et al., 1993; Zhou and Kuiken, 2006; Marker and Maluf, 2014). The method proposed in the current study aims to extract LFPs in the ECG-contaminated frequency band, i.e., the low-frequency band. Thus, a 23 Hz tone should be sufficient for validation of the method. Two additional Ag/Cl disc-electrodes beside the titanium case delivered a standard ECG waveform generated from a digital function generator. The period of the ECG signal was set to 750 ms.

#### Stimulation and Recording

Electrode contacts 1 and 3 were configured as the bipolar montage for LFP recording. The sensing-enabled neurostimulator case and electrode contact 2 were configured as the monopolar montage for ECG reference recording. The bipolar and monopolar recordings were synchronized by the sensing-enabled neurostimulator. The sampling rate was 1,000 Hz. Monopolar stimulation was delivered between the case and contact 2 in the DBS electrodes. In a real recording scenario after the implantation of the sensing-enabled neurostimulators, no power line noise could be sensed. However, in the saline simulation, strong power line noises could be sensed by the neurostimulator. To avoid cross-interference (differential harmonics) between stimulation and the power frequency (50 Hz), the stimulation frequency was set to 150 Hz. The stimulation pulse width was 60 μs. To study the effects of DBS artifacts, the stimulation amplitude was set to off, 0.5, 1.0, 1.5, 2.0, 2.5, and 3.0 V. A segment of clean LFP was recorded after the ECG source and stimulation were both turned off. Under each condition, data was recorded for at least 300 s.

### Data Preprocessing

All recordings were first filtered by a 1 Hz high-pass filter (digital Butterworth) to suppress the baseline fluctuations and then filtered by a 470 Hz low-pass filter (digital Butterworth) to exclude higher aliasing frequencies. For recordings of a clean LFP, the power frequency of 50 Hz and its harmonics at 100 Hz were filtered by an adaptive trap filter (Keshtkaran and Yang, 2014). For the recordings of ECG-contaminated LFP, DBS artifacts combined with the power frequency inferences were filtered out. To retain the information in the frequency domain as much as possible, we used a custom-designed adaptive trap filter to remove the DBS artifacts. For recordings of the ECG reference signal, data was filtered by the adaptive filter used for the power frequency and then filtered by a 100 Hz low-pass filter (3rd-order digital Butterworth).

### ECG Artifact Removal

In this study, a monopolar channel was added for ECG recording. We took advantage of two pre-existing methods, i.e., template subtraction and adaptive filtering, to evaluate the effectiveness of ECG artifact removal. MATLAB (version 2016a) was used for all the calculations.

#### Template Subtraction

The template subtraction method extracts an ECG artifact template from the contaminated LFP recordings and subtracts the template in each ECG artifacts spike. The method mainly involved three steps: (1) ECG spike detection, (2) ECG artifact template extraction, and (3) ECG artifact template subtraction. For ECG spike detection, we applied an accurate QRS complex detector proposed by Pan and Tompkins (1985) and Hamilton and Tompkins (1986) to the ECG reference channel. The detected R-waves were used to align the ECG artifacts in the contaminated LFPs. A template was extracted by averaging the ECG artifacts and filtered by a 100 Hz low-pass filter (3rd-order digital Butterworth). The filtered template was then subtracted from the ECG-contaminated LFPs.

#### Adaptive Filtering

The general methodology of adaptive filtering is to estimate the ECG artifacts from the contaminated LFPs by minimizing the errors between the output and the ECG reference. In this study, we took advantage of the built-in frequency domain adaptive filter toolbox (dsp.FrequencyDomainAdaptiveFilter) in MATLAB to design the adaptive filter. The length of the coefficient vector of the adaptive filter was 64 and the block length of the coefficients updates was 1,000. The frequency-domain adaptive filter iterated the coefficients vector after transformation of the inputs to the frequency domain using discrete Fourier transforms. For detailed algorithms, please see Shynk (1992). The input was the ECG reference and the desired signal was the contaminated LFP. The outputs were the estimated ECG artifacts. The estimated ECG artifacts were then subtracted from the contaminated LFP recordings.

### Performance Evaluation

Power spectral density (PSD) was estimated by the Welch’s method. The frequency resolution of the PSDs was 1 Hz and the overlap of the Hamming window was 50%. Logarithmic PSDs were plotted for comparison between the clean LFP, the contaminated LFPs, and the cleaned LFPs. To quantitatively evaluate artifact removal, the signal-noise-ratio (SNR) and the root mean square logarithmic error (RMSLE) of the PSDs were calculated.

The SNR was defined as the ratio of the average power in the signal and noise bands. The SNR was calculated as:

(1) $$S\,N\,R=10\,{\text{log}}_{10}\,\frac{P\,o\,w\,e\,r_{s\,i\,g\,n\,a\,l}}{P\,o\,w\,e\,r_{n\,o\,i\,s\,e}}$$

where *Power*_*signal*_ represents the average power of the signal in the band of 22–23 Hz, *Power*_*noise*_ represents the average power of the ECG contamination in the band of 1–100 Hz, excluding 22–23 Hz. A larger SNR indicates better ECG artifact removal.

Because the spectrum was not uniformly distributed across the band, we used the RMSLE to evaluate the difference between the PSDs of cleaned LFPs and the simulated clean LFP. By logarithmic transformation, RMSLE evaluates the differences between small values (such as the power of the noise) similarly to differences between big values (such as the power of the signal). The RMSLE was calculated as:

(2) $$R\,M\,S\,L\,E=\sqrt{\frac{1}{N}\,\sum_{f_{1}}^{f_{2}}{\left(p\,s\,d_{s\,i\,n}\,\left(f\right)-p\,s\,d\,\left(f\right)\right)}^{2}}$$

where *psd_*si*__*n*_(f)* represents the logarithmic PSD of the clean LFP, *psd(f)* represents the logarithmic PSD of the contaminated or cleaned LFPs. f_1_ and f_2_ indexes the relevant band for analysis, which is 1–100 Hz (i.e., *N* = 100). A smaller RMSLE indicates better performance of ECG artifact removal.

## Results

### Simulated LFP Recordings

**FIGURE 4:**
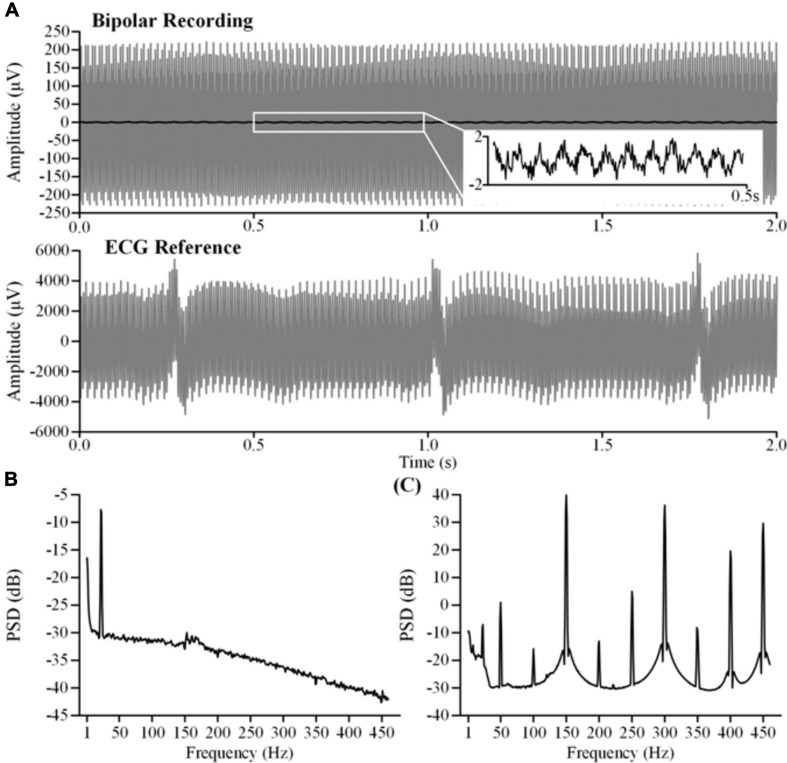
Characteristics of the original recordings. **(A)** The top axis is the bipolar recording of the clean LFP (dark line) and the contaminated LFP (gray line). The bottom axis is the monopolar recording of theECG reference. The contaminated LFP and the ECG reference were synchronously recorded during stimulation (2.5 V, 150 Hz, 60μs). **(B)** The PSD of the clean LFP. **(C)** The PSD of the contaminated LFP.

In this study, we synchronously recorded bipolar and monopolar channels for ECG artifact removal. Examples of the recordings are shown in Figure 4A. The top axis in Figure 4A is the time series of bipolar recordings. The dark line is the clean LFP and the gray line is the contaminated LFP. The residual DBS (2.5 V, 150 Hz, 60 μs) artifacts in the contaminated LFP are more than two orders of magnitude greater than in the clean LFP. The ECG artifacts could hardly be distinguished in the time series. The bottom axis in Figure 4A is the time series of the monopolar recording synchronized with the contaminated LFP. Although the DBS artifacts were greater in the monopolar recording, a clear ECG waveform could be distinguished. Figure 4B shows the PSD of the clean LFP. Figure 4C shows the PSD of the contaminated LFP. The fundamental frequency of the DBS artifacts was 150 Hz and the harmonic frequencies were 300 and 450 Hz. Other aliasing frequencies superposed with the power frequency were integer multiples of 50 Hz.

### ECG Artifact Removal

#### ECG Recording Performance

**FIGURE 5:**
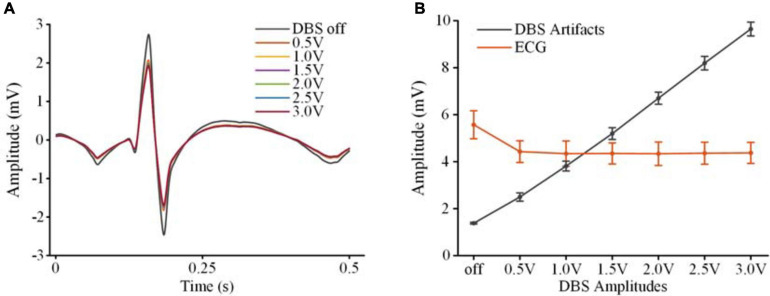
Extraction of ECG spikes from monopolar recordings. **(A)** The averaged ECG spikes in DBS off and DBS on states. **(B)** The peak-to-peak values of the ECG spikes and DBS artifacts. The dots are mean values and the error bars are standard deviations.

To extract clear ECG spikes, monopolar recordings were filtered by a fixed 100 Hz low-pass filter (3rd-order digital Butterworth). To study whether the ECG spikes in the monopolar recordings could be consistently restored in the presence of different DBS artifacts, we compared the amplitudes of the extracted spikes. The spikes were aligned according to the detected R-waves. Figure 5A shows the averaged spikes extracted under different stimulation amplitudes. Figure 5B shows the peak-to-peak amplitudes of each ECG spike and the peak-to-peak amplitudes of the DBS artifacts in the original monopolar recordings. Compared with the mean value in the DBS off state (5.57 ± 0.60 mV, mean ± SD), the amplitudes of the spikes decreased slightly in the DBS on state (4.54 ± 0.66 mV, *p* < 0.001, *N* = 266). Although the amplitudes of the DBS artifacts increased from 2.50 ± 1.8 mV (0.5 V DBS) to 9.65 ± 0.29 mV (3.0 V DBS), no significant changes were found between the amplitudes of the ECG spikes (*p* > 0.064, *N* = 266). The results indicated that by using a simple fixed 100 Hz low-pass filter, ECG spikes could be extracted from the monopolar recordings under different stimulation amplitudes. The robust performance of spike extraction provided a foundation for ECG artifact removal.

#### Performance of ECG Artifact Removal

To validate the feasibility of using the monopolar channel as a reference for ECG artifact removal, we tested two pre-existing methods, i.e., template subtraction and adaptive filtering. Performance of ECG artifact removal using each method was evaluated in the DBS off and on states.

**FIGURE 6:**
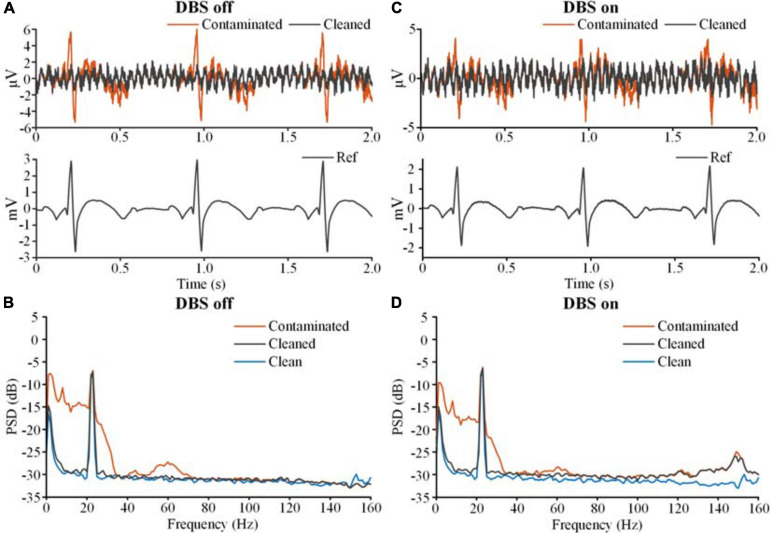
The results of ECG artifact removal using template subtraction. **(A)** The results of ECG removal with the DBS off. The bipolar recordings before (contaminated, red line) and after (cleaned, gray line) ECG removal were plotted on the top axis. The monopolar recording of the ECG reference was plotted on the bottom axis. **(B)** The PSDs of the bipolar recording before and after ECG removal. The blue line is the PSD of the clean LFP. **(C)** The results of ECG removal fromthe recording with the DBS on. **(D)** The PSDs of the bipolar recordings before and after ECG removal.

Figure 6 shows the results of template subtraction. In Figure 6A, the red line on the top axis is the ECG-contaminated LFP recorded in the DBS off state and the gray line is the cleaned LFP. The bottom axis shows the distinct R-waves in the synchronized monopolar channel. Using the monopolar channel as an ECG reference, the template subtraction method removed the ECG artifacts in the contaminated LFP. Figure 6B shows the PSDs of the ECG-contaminated LFP (red line) and cleaned LFP (gray line). The ECG artifacts mainly contaminated the band below 100 Hz. After removing the artifacts, the PSD of the cleaned LFP was overlaid with the PSD of the simulated clean LFP (blue line). In Figure 6C, an ECG-contaminated LFP recorded in the DBS on state (2.5 V, 150 Hz, 60 μs) and the corresponding cleaned LFP are plotted on the top axis. After removing the DBS artifacts, the monopolar recording reflected distinct R-waves (bottom axis). Using the monopolar channel as an ECG reference, the ECG artifacts were successfully removed. Figure 6D shows the PSDs of the contaminated (red line) and cleaned (gray line) LFPs in the DBS on state. The ECG-contaminated band below 100 Hz was restored in the cleaned LFP. We found a slightly elevated noise floor in the PSD of the cleaned LFP, which partially resulted from the ECG signal generator. In addition, some noise came from residual stimulation artifacts. Although the stimulation artifacts were largely removed, some harmonics and aliasing components remained, such as at 250, 300, 400, and 450 Hz. Spectrum estimation of these residual stimulation artifacts would unavoidably introduce leakage of the power to a broadband range.

**FIGURE 7:**
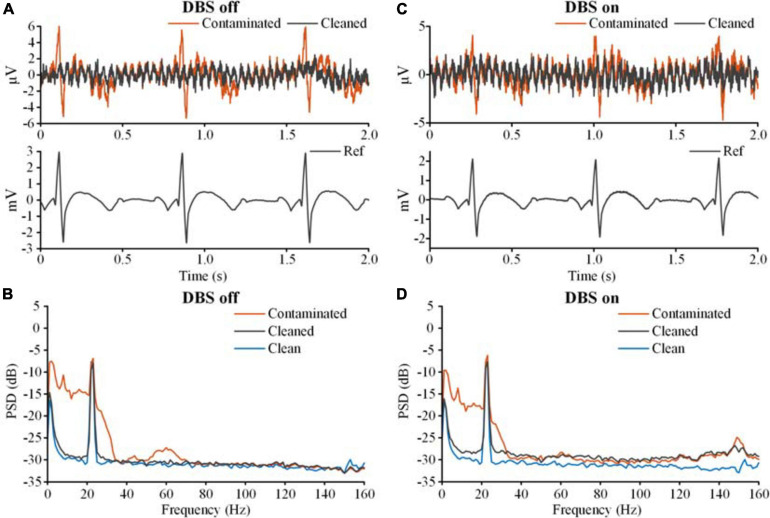
The results of ECG artifact removal using adaptive filtering. **(A)** The results of ECG removal of the recording with the DBS off. The bipolar recordings before (contaminated, red line) and after (cleaned, gray line) ECG removal were plotted on the top axis. The monopolar recording of the ECG reference was plotted on the bottom axis. **(B)** The PSDs of the bipolar recording before and after ECG removal. The blue line is the PSD of the clean LFP. **(C)** The results of ECG removal from the recording with the DBS on. **(D)** The PSDs of the bipolar recordings before and after ECG removal.

Figure 7 shows the results of adaptive filtering. Figures 7A,B show recordings in the DBS off state, and Figures 7C,D show recordings in the DBS on state (2.5 V, 150 Hz, 60 μs). The ECG-contaminated band below 100 Hz was restored in the cleaned LFPs. Therefore, both template subtraction and adaptive filtering could remove the ECG artifacts.

To quantitatively evaluate the performance of the method, SNRs and RMSLEs of the PSDs were calculated. Table 1 shows the SNR and RMSLE of the cleaned LFPs. Compared with the ECG-contaminated LFPs, the SNRs of the cleaned LFPs were greatly improved, with values close to the SNR of the clean LFP (20.87 dB). The RMSLEs also decreased, indicating that the differences in the PSDs between the cleaned LFP and the simulated clean LFP decreased after ECG artifact removal. Neither SNR nor RMSLE were significantly influenced by increasing the stimulation amplitude.

**TABLE 1:** Performance evaluation using signal-noise-ratio (SNR) and root mean square logarithmic error (RMSLE).

**Stimulation^#^(V)**	**SNR (dB)**		**RMSLE (dB)**	
	
	TS*	AF*	TS*	AF*
Off	19.88 (8.93)	19.16 (8.22)	0.94 (−6.60)	0.94 (−6.59)
0.5	18.97 (5.89)	18.91 (5.82)	1.58 (−5.09)	1.37 (−5.30)
1.0	20.69 (7.17)	19.64 (6.11)	1.02 (−5.33)	1.26 (−5.09)
1.5	20.57 (6.92)	19.25 (5.61)	1.10 (−5.14)	1.34 (−4.90)
2.0	20.48 (6.73)	19.60 (5.85)	1.14 (−5.01)	1.35 (−4.80)
2.5	20.52 (6.58)	19.14 (5.19)	1.17 (−4.87)	1.85 (−4.20)
3.0	20.53 (6.41)	19.35 (5.23)	1.32 (−4.64)	1.60 (−4.36)

**The values in the brackets are the differences with the contaminated local field potentials (LFPs). ^#^Thedeep brain stimulation (DBS) artifacts in the recordings were removed. ^∗^TS, template subtraction; AF, adaptive filtering.**

### Performance Under Residual DBS Artifacts

In section “ECG Artifact Removal,” the DBS artifacts under different stimulation amplitudes were precisely filtered. However, due to complex recording conditions and various filtering methods, DBS artifacts could remain in the LFPs at varying levels. Therefore, it is crucially important to evaluate the performance in a background of residual DBS artifacts.

**FIGURE 8:**
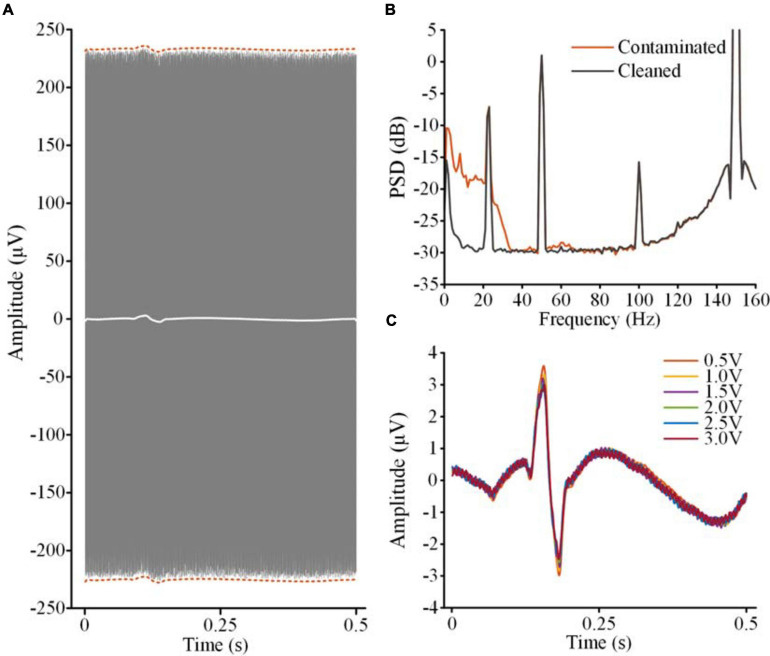
ECG artifact removal without extra filtering of DBS artifacts. **(A)** The gray lines are the overlapped contaminated LFPs aligned according to the R-waves detected in the monopolar channel. The dashed red lines indicate the envelope of the ECG artifacts. The white line is the averaged waveform of the aligned recordings. **(B)** PSDs of the contaminated LFP and ECG-cleaned LFP. **(C)** The templates of the ECG artifacts in the contaminated LFPs with different DBS amplitudes.

Figure 8A shows the contaminated LFP in the stimulation on state (gray lines, 2.5 V, 150 Hz, 60 μs) without filtering DBS artifacts. The recordings were aligned according to the ECG spikes detected in the monopolar channel. By overlapping the recordings, the envelope of the ECG artifacts could be distinguished (dashed red lines). The white line in Figure 8A shows the average waveform of the overlapped recordings. Using this template, the ECG artifacts were subtracted from the contaminated LFP. Figure 8B shows the PSDs of the contaminated LFP (red line) and ECG-cleaned LFP (gray line). The ECG-contaminated band was restored while the DBS artifacts remained. Figure 8C shows the ECG templates extracted from the contaminated LFPs. The performance of template extraction was stable across 0.5–3.0 V DBS without additional filtering of the DBS artifacts.

Table 2 shows a quantitative evaluation of the ECG removal performance without filtering the DBS artifacts. To exclude the effects of increasing the DBS artifacts, SNR and RSMLE were calculated for the band below 40 Hz. Compared with the ECG-contaminated LFPs, both the SNR and RMSLE of the cleaned LFPs were improved. SNR of the cleaned LFPs was close to the value of the simulated clean LFP (18.70 dB). From 1.0 V DBS to 3.0 V DBS, the SNR of the cleaned LFPs decreased from 18.29 to 17.62 dB and the RSMLE increased from 0.98 to 1.9 dB, indicating an attenuation of performance. However, this attenuation of the performance was very slight compared with the increase on the DBS artifacts from 1.0 V DBS (211.01 μV) to 3.0 V DBS (516.18 μV). The results indicate that the ECG artifacts in the contaminated LFPs can be suppressed to a significant extent even in the presence of residual DBS artifacts.

**TABLE 2:** Performance evaluation of electrocardiogram (ECG) artifact removal without extra filtering deep brain stimulation (DBS) artifacts.

**Stimulation (V)**	**SNR (dB)**	**RMSLE (dB)**
0.5	16.02 (6.77)	1.74 (−8.01)
1.0	18.29 (8.58)	0.98 (−8.35)
1.5	18.01 (8.17)	1.10 (−8.02)
2.0	17.75 (7.81)	1.29 (−7.66)
2.5	17.82 (7.73)	1.32 (−7.50)
3.0	17.62 (7.30)	1.90 (−6.77)

**Signal-noise-ratio (SNR) androot mean square logarithmic error (RMSLE) were calculated for the band below 40 Hz. The values in the brackets are the differences with the contaminated local field potentials(LFPs).**

## Discussion

In this study, we proposed an approach to removing ECG artifacts from the signals recorded by sensing-enabled neurostimulators. A simultaneous monopolar montage was added and distinct R-waves could be recorded in the time series. Using the monopolar recording as an ECG reference, conventional ECG filtering methods, including template subtraction and adaptive filtering, could effectively remove ECG artifacts in the bipolar LFP recordings. The performance of this method was not significantly influenced by the magnitude of residual DBS artifacts. Combining the monopolar and bipolar recordings, the method could remove ECG artifacts without additional complex signal processing.

### Using Simultaneous Monopolar Recording as an ECG Reference

Removing ECG artifacts is a common challenge in the field of electrophysiological signal processing. There have been many algorithms proposed in previous studies, such as template subtraction (Zhou et al., 2007; Marker and Maluf, 2014), adaptive filtering (Lu et al., 2009; Sweeney et al., 2012), ICA (Mak et al., 2010), and other blind-source separation methods (Sweeney et al., 2012). Most of the methods proved effective for multi-channel EEG and electromyography recording. Some methods were specifically designed for single-channel recording (Sweeney et al., 2012). However, most of the pre-existing methods cannot be directly applied to the signals recorded by implanted sensing-enabled neurostimulators. For sensing-enabled neurostimulators, the electrophysiological signals (LFPs) are usually independently recorded without ECG reference. Thus, the methods of template subtraction or adaptive filtering, both of which require reference signals, are not suitable. The number of synchronized channels of sensing-enabled neurostimulators is usually very limited. For recording in the DBS on state, the sensing-enabled DBS usually records only a few synchronized channels due to limited electrodes. Thus, the method of ICA which needs multi-channel recordings is not fully applicable to the LFPs recorded during DBS. Although there are some single-channel blind-source separation methods (Sweeney et al., 2012), the cost of the algorithms is usually higher and the robustness of the performance is inconclusive due to residual DBS artifacts. Lack of an ECG reference and recording channels makes it difficult to remove ECG artifacts in the LFPs recorded by sensing-enabled neurostimulators.

There are two kinds of recording montages in sensing-enabled neurostimulators, i.e., the bipolar montage and the monopolar montage. The bipolar montage differentiates the potentials between pairs of contacts in the DBS electrode while the monopolar montage differentiates the potentials between one contact in the DBS electrode and the titanium case of the neurostimulator (Stanslaski et al., 2012; Qian et al., 2014, 2017). Previous studies reported that the monopolar montage is considerably affected by remote volume conductivity (Marmor et al., 2017). Thus, it is possible to record ECG references using a monopolar montage.

In this study, we demonstrated the feasibility of recording ECG reference signals using the monopolar montage of a sensing-enabled neurostimulator. Our results showed that although the DBS artifacts were large, a clear R-waveform could be extracted in the monopolar channel. However, the peak-to-peak amplitudes of ECG artifacts in bipolar recordings *in vivo* might be unstable due to respiration or motion. Previous literature reported that respiration or even relatively slow motion over time would induce baseline wander in the ECG signals (Satija et al., 2018; Chatterjee et al., 2020). The peak-to-peak amplitudes of ECG spikes vary by 15% due to baseline wander (Friesen et al., 1990; Satija et al., 2018). For rapid motion artifacts, the effect is usually over a short duration and the fluctuation could be 500 percent of the peak-to-peak amplitudes of ECG spikes (Friesen et al., 1990; Satija et al., 2018). In Figure 6C, the coefficient of variation of the ECG peak-to-peak amplitudes in bipolar recordings was 13.07% (std/mean) and the value of the ECG spikes in monopolar recordings was 0.7%. To estimate the influence of these variations, we added amplitude-modulated white noise to the recordings. The coefficients of variation increased to 21.62% in bipolar recordings and 16.37% in monopolar recordings. Both variations in the bipolar and monopolar recordings were above the typical value reported in the literature. The methods of template subtraction and adaptive filtering were applied to remove ECG-artifacts in the noisy recordings. The results showed that the ECG artifacts could still largely be removed (see Supplementary Material for details).

The primary benefit of the current study is to make conventional ECG removal methods that require ECG reference signals applicable to LFPs recorded by sensing-enabled neurostimulators. The method proposed in our study may help researchers to extract real LFPs to study chronic deep brain neural activities and DBS mechanisms. Most such research is conducted retrospectively. Where real-time analysis is needed, such as in closed-loop stimulation, a template could only be extracted in a short window. In a previous study using the template subtraction method for real-time ECG artifact removal, 30 ECG spikes were used to update the template (Abbaspour and Fallah, 2014). We tested time windows of 30, 20, and 10 s, respectively, and found that the template was valid in the 30 s window (see Supplementary Material for details). Because the interval of the simulated ECG spikes was 0.75 s, the results indicate that at least 40 ECG spikes would be necessary for extracting a valid template. In real-time scenarios, the templates could be accumulated and the performance could be improved by introducing overlap and padding techniques for each window. Alternatively, we could use the method of adaptive filtering for real-time analysis. Future studies should focus on development and refinement of detailed real-time algorithms based on specific real-time scenarios.

### Effects of DBS Artifacts

The magnitudes of the stimulation pulses in the monopolar recording chain were asymmetric. The asymmetry would induce large DBS artifacts in the ECG reference channel. Consequently, different magnitudes of residual DBS artifacts might influence detection of ECG spikes. Previous studies have shown that as long as the amplifiers are not saturated, the real signals in an asymmetric montage can still be restored (Kent and Grill, 2012; Qian et al., 2017). In our study, we compared the amplitudes of the ECG spikes under different DBS amplitudes. The results showed that although the DBS artifacts increased greatly from 0.5 V DBS to 3.0 V DBS, no significant difference was found between the amplitudes of extracted ECG spikes. Robust R-waves could be extracted from the monopolar recordings. This result provided the foundation for the removal of ECG artifacts.

In the bipolar channel of the sensing-enabled neurostimulator, the amplitudes of DBS artifacts could be more than two orders of magnitude greater than the ECG artifacts and LFP signals. In our study, we precisely filtered the DBS artifacts to restore the time series. However, residual DBS artifacts were usually unavoidable in LFP recordings due to complex recording conditions and different filtering methods. To evaluate the effects of residual DBS artifacts on the removal of ECG artifacts, we studied the performance of the method without extra filtering of the DBS artifacts. The results showed that even in the presence of large DBS artifacts, ECG artifacts were suppressed significantly, indicating that the method was not sensitive to residual DBS artifacts. This finding emphasizes the robustness of the method and limits restrictions on its algorithms.

### Limitations and Future Work

Removal of ECG artifacts in our study was only tested using the conventional fixed-template subtraction method and a simple adaptive filter. The performance could be improved by optimizing the extraction template and design of the adaptive filter. The current method was only tested on simulated signals recorded in saline solution. Although we have tested and verified the methods using physiological ECG levels, the performance of the method could still be influenced by chronic changes in the recording impedance, leakage of fluid, and different *in-vivo* electromagnetic environments. The performance needs to be confirmed for real *in-vivo* recordings in future studies. In addition, we should estimate whether the algorithms can be integrated into an embedded system. The goal of the current study was to provide an ECG reference for use with current methods and thus to reduce the complexity of the related algorithms. Based on our survey of the computation efficiency and storage resource of the current sensing-enabled neurostimulators, both of the approaches proposed in this study could be completed within 400–500 ms. This is sufficient for real-time analysis. Thus, this method holds potential for development of algorithms for real-time removal of ECG artifacts in an embedded system. However, there is still plenty of work that needs to be done in future embedded development, especially for addressing the trade-off between power consumption and analytical performance in specific applications.

## Data Availability Statement

The raw data supporting the conclusions of this article will be made available by the authors, without undue reservation, to any qualified researcher.

## Author Contributions

YC, BM, and LL designed the research. YC conducted the research, collected the data, and wrote the initial manuscript. BM, HH, and LL revised the manuscript. All authors read and approved the final manuscript.

## Conflict of Interest

LL, HH, and BM serve on the scientific advisory board for Beijing Pins Medical Co., Ltd., and were listed as inventors in issued patents and patent applications on the deep brain stimulator used in this work. The remaining author declares that the research was conducted in the absence of any commercial or financial relationships that could be construed as a potential conflict of interest.

## References

[B1] Abbaspour S. Fallah A. (2014). Removing ECG artifact from the surface EMG signal using adaptive subtraction technique. *J. Biomed. Phys. Eng. Anal.* 4 33–38. PMC4258854 25505766

[B2] Anidi C. O’Day J. J. Anderson R. W. Afzal M. F. Syrkin-Nikolau J. Velisar A. (2018). Neuromodulation targets pathological not physiological beta bursts during gait in Parkinson’s disease. *Neurobiol. Dis.* 120 107–117. 10.1016/j.nbd.2018.09.004 30196050 PMC6422345

[B3] ASTM (2011). *F2182-11a: Standard Test Method for Measurement of Radio Frequency Induced Heating on or Near Passive Implants During Magnetic Resonance Imaging.* West Conshohocken, PA: ASTM International.

[B4] Canessa A. Pozzi N. G. Arnulfo G. Brumberg J. Reich M. M. Pezzoli G. (2016). Striatal dopaminergic innervation regulates subthalamic beta-oscillations and cortical-subcortical coupling during movements: preliminary evidence in subjects with Parkinson’s disease. *Front. Hum. Neurosci.* 10:611. 10.3389/fnhum.2016.00611 27999534 PMC5138226

[B5] Chatterjee S. Thakur R. S. Yadav R. N. Gupta L. Raghuvanshi D. K. (2020). Review of noise removal techniques in ECG signals. *IET Signal Process.* 14 569–590. 10.1049/iet-spr.2020.0104

[B6] Friesen G. M. Jannett T. C. Jadallah M. A. Yates S. L. Quint S. R. Nagle H. T. (1990). A comparison of the noise sensitivity of nine QRS detection algorithms. *IEEE Trans. Biomed. Eng.* 37 85–98. 10.1109/10.43620 2303275

[B7] Golshan H. M. Hebb A. O. Mahoor M. H. (2020). LFP-Net: a deep learning framework to recognize human behavioral activities using brain STN-LFP signals. *J. Neurosci. Methods* 335:108621. 10.1016/j.jneumeth.2020.108621 32027889

[B8] Hamilton P. S. Tompkins W. J. (1986). Quantitative investigation of QRS detection rules using the MIT/BIH arrhythmia database. *IEEE Trans. Biomed. Eng.* 33 1157–1165. 10.1109/TBME.1986.325695 3817849

[B9] Hell F. Plate A. Mehrkens J. H. Bötzel K. (2018). Subthalamic oscillatory activity and connectivity during gait in Parkinson’s disease. *NeuroImage Clin.* 19 396–405. 10.1016/j.nicl.2018.05.001 30035024 PMC6051498

[B10] Kent A. Grill W. (2012). Recording evoked potentials during deep brain stimulation: development and validation of instrumentation to suppress the stimulus artefact. *J. Neural Eng.* 9:036004. 10.1088/1741-2560/9/3/036004 PMC3360802 22510375

[B11] Keshtkaran M. R. Yang Z. (2014). A fast, robust algorithm for power line interference cancellation in neural recording. *J. Neural Eng.* 11:026017. 10.1088/1741-2560/11/2/026017 24658388

[B12] Lu G. Brittain J.-S. Holland P. Yianni J. Green A. L. Stein J. F. (2009). Removing ECG noise from surface EMG signals using adaptive filtering. *Neurosci. Lett.* 462 14–19. 10.1016/j.neulet.2009.06.063 19559751

[B13] Mak J. N. Hu Y. Luk K. D. K. (2010). An automated ECG-artifact removal method for trunk muscle surface EMG recordings. *Med. Eng. Phys.* 32 840–848. 10.1016/j.medengphy.2010.05.007 20561810

[B14] Marker R. J. Maluf K. S. (2014). Effects of electrocardiography contamination and comparison of ECG removal methods on upper trapezius electromyography recordings. *J. Electromyogr. Kinesiol.* 24 902–909. 10.1016/j.jelekin.2014.08.003 25200192 PMC9321361

[B15] Marmor O. Valsky D. Joshua M. Bick A. S. Arkadir D. Tamir I. (2017). Local vs. volume conductance activity of field potentials in the human subthalamic nucleus. *J. Neurophysiol.* 117 2140–2151. 10.1152/jn.00756.2016 28202569 PMC5454468

[B16] Martini M. L. Oermann E. K. Opie N. L. Panov F. Oxley T. Yaeger K. (2020). Sensor modalities for brain-computer interface technology: a comprehensive literature review. *Neurosurgery* 86 E108–E117. 10.1093/neuros/nyz286 31361011

[B17] Meidahl A. C. Tinkhauser G. Herz D. M. Cagnan H. Debarros J. Brown P. (2017). Adaptive deep brain stimulation for movement disorders: the long road to clinical therapy. *Mov. Disord.* 32 810–819. 10.1002/mds.27022 28597557 PMC5482397

[B18] Neumann W. J. Staub Bartelt F. Horn A. Schanda J. Schneider G.-H. Brown P. (2017). Long term correlation of subthalamic beta band activity with motor impairment in patients with Parkinson’s disease. *Clin. Neurophysiol.* 128 2286–2291. 10.1016/j.clinph.2017.08.028 29031219 PMC5779610

[B19] Pan J. Tompkins W. J. (1985). A real-time QRS detection algorithm. *IEEE Trans. Biomed. Eng.* 32 230–236. 10.1109/TBME.1985.325532 3997178

[B20] Qian X. Chen Y. Feng Y. Ma B. Hao H. Li L. (2017). A method for removal of deep brain stimulation artifact from local field potentials. *IEEE Trans. Neural Syst. Rehabil. Eng.* 25 2217–2226. 10.1109/TNSRE.2016.2613412 28113981

[B21] Qian X. Hao H. Ma B. Wen X. Hu C. Li L. (2014). Implanted rechargeable electroencephalography (EEG) device. *Electron. Lett.* 50 1419–1421. 10.1049/el.2014.1820

[B22] Quinn E. J. Blumenfeld Z. Velisar A. Koop M. M. Shreve L. A. Trager M. H. (2015). Beta oscillations in freely moving Parkinson’s subjects are attenuated during deep brain stimulation. *Mov. Disord.* 30 1750–1758. 10.1002/mds.26376 26360123

[B23] Ramirez-Zamora A. Giordano J. Gunduz A. Alcantara J. Cagle J. N. Cernera S. (2020). Proceedings of the seventh annual deep brain stimulation think tank: advances in neurophysiology, adaptive DBS, virtual reality, neuroethics and technology. *Front. Hum. Neurosci.* 14:54. 10.3389/fnhum.2020.00054 32292333 PMC7134196

[B24] Redfern M. S. Hughes R. E. Chaffin D. B. (1993). High-pass filtering to remove electrocardiographic interference from torso EMG recordings. *Clin. Biomech.* 8 44–48. 10.1016/S0268-0033(05)80009-9 23915829

[B25] Satija U. Ramkumar B. Manikandan M. S. (2018). A review of signal processing techniques for electrocardiogram signal quality assessment. *IEEE Rev. Biomed. Eng.* 11 36–52. 10.1109/RBME.2018.2810957 29994590

[B26] Shen H. (2014). Neuroscience: tuning the brain. *Nature* 507 290–292. 10.1038/507290a 24646978

[B27] Shynk J. J. (1992). Frequency-domain and multirate adaptive filtering. *IEEE Signal Process. Mag.* 9 14–37. 10.1109/79.109205

[B28] Sorkhabi M. M. Benjaber M. Brown P. Denison T. (2020). “Physiological artifacts and the implications for brain-machine-interface design,” in *Proceedings of the 2020 IEEE International Conference on Systems, Man, and Cybernetics (SMC)*, (Toronto, ON: IEEE), 1498–1498. 10.1109/SMC42975.2020.9283328 PMC7116608 33479560

[B29] Stanslaski S. Afshar P. Cong P. Giftakis J. Stypulkowski P. Carlson D. (2012). Design and validation of a fully implantable, chronic, closed-loop neuromodulation device with concurrent sensing and stimulation. *IEEE Trans. Neural Syst. Rehabil. Eng.* 20 410–421. 10.1109/TNSRE.2012.2183617 22275720

[B30] Swann N. C. de Hemptinne C. Miocinovic S. Qasim S. Ostrem J. L. Galifianakis N. B. (2018). Chronic multisite brain recordings from a totally implantable bidirectional neural interface: experience in 5 patients with Parkinson’s disease. *J. Neurosurg.* 128 605–616. 10.3171/2016.11.JNS161162 28409730 PMC5641233

[B31] Sweeney K. T. Ward T. E. McLoone S. F. (2012). Artifact removal in physiological signals—Practices and possibilities. *IEEE Trans. Inform. Technol. Biomed.* 16 488–500. 10.1109/TITB.2012.2188536 22361665

[B32] Trager M. H. Koop M. M. Velisar A. Blumenfeld Z. Nikolau J. S. Quinn E. J. (2016). Subthalamic beta oscillations are attenuated after withdrawal of chronic high frequency neurostimulation in Parkinson’s disease. *Neurobiol. Dis.* 96 22–30. 10.1016/j.nbd.2016.08.003 27553876

[B33] Vansteensel M. J. Pels E. G. Bleichner M. G. Branco M. P. Denison T. Freudenburg Z. V. (2016). Fully implanted brain–computer interface in a locked-in patient with ALS. *N. Engl. J. Med.* 375 2060–2066. 10.1056/NEJMoa1608085 27959736 PMC5326682

[B34] Velisar A. Syrkin-Nikolau J. Blumenfeld Z. Trager M. Afzal M. Prabhakar V. (2019). Dual threshold neural closed loop deep brain stimulation in Parkinson disease patients. *Brain Stimul.* 12 868–876. 10.1016/j.brs.2019.02.020 30833216

[B35] Zhou P. Kuiken T. A. (2006). Eliminating cardiac contamination from myoelectric control signals developed by targeted muscle reinnervation. *Physiol. Meas.* 27 1311–1327. 10.1088/0967-3334/27/12/005 17135702

[B36] Zhou P. Lock B. Kuiken T. A. (2007). Real time ECG artifact removal for myoelectric prosthesis control. *Physiol. Meas.* 28 397–413. 10.1088/0967-3334/28/4/006 17395995

