# Supplementary material for: Removal of Electrocardiogram Artifacts From Local Field Potentials Recorded by Sensing-Enabled Neurostimulator
Source: Front Neurosci. 2021 Apr 12;15:637274. doi: 10.3389/fnins.2021.637274 (PMC8071948; doi:10.3389/fnins.2021.637274)
Supplement: Supplementary file 1 [file Table_1.DOCX]

Supplementary Material

# The estimation of the varies of the ECG peak-to-peak amplitudes

To estimate the influence of the varies of the ECG peak-to-peak amplitude, we added amplitude-modulated white noises to the recordings. As shown in Supplementary Figure 1, the coefficient of variation of the ECG peak-to-peak amplitudes in bipolar recordings increased to 21.62% (std/mean), and the value of the ECG spikes in monopolar recordings increased to 16.37%. Both the varies in the bipolar and monopolar recordings were above the typical value reported in the literature. Supplementary Figure 2 shows the PSDs of the data before and after the ECG artifacts were removed.


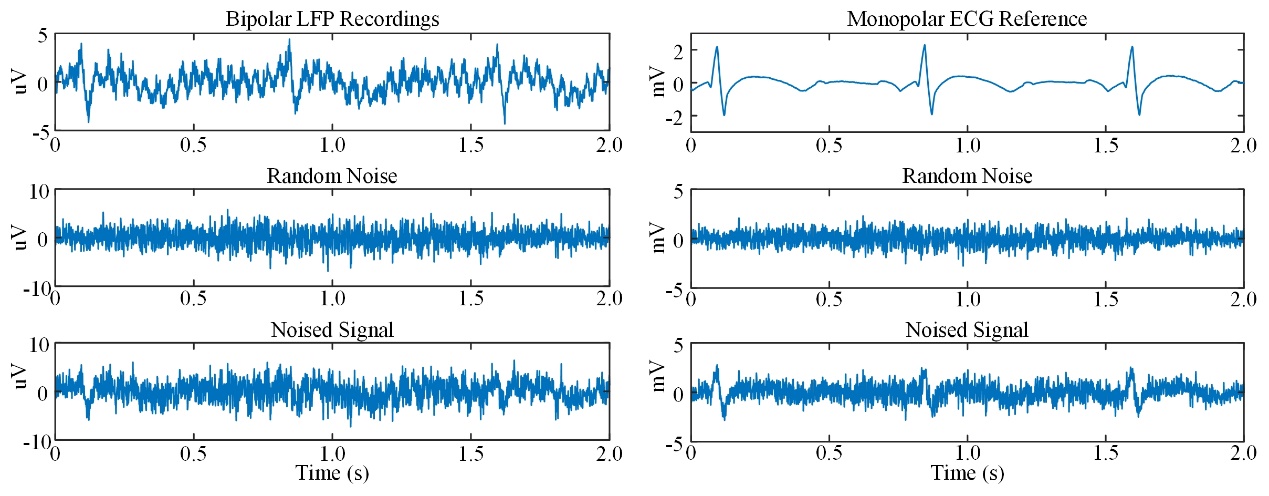


**Supplementary Figure 1.** Random noises were added to the recordings to increase the varies of the peak-to-peak amplitude of ECG spikes.


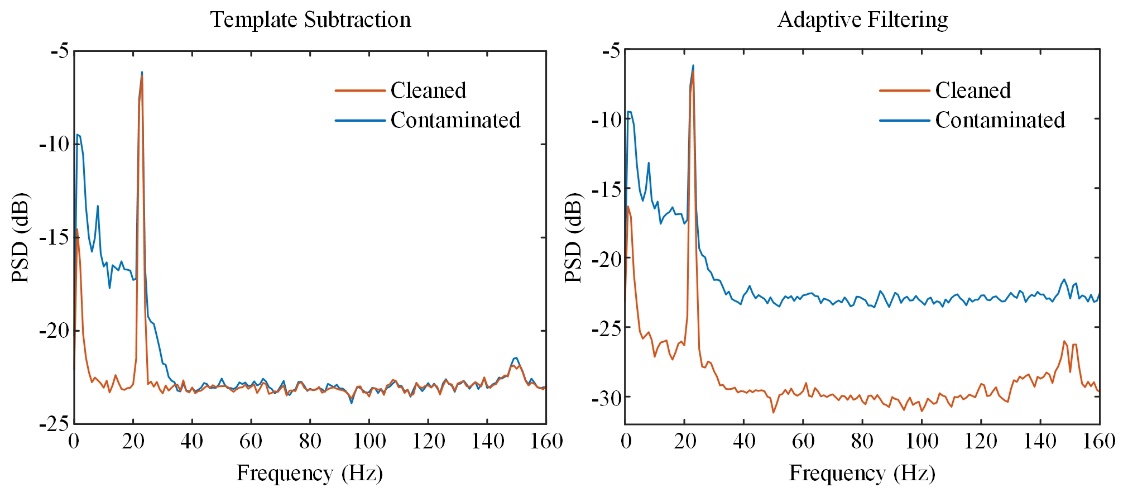


**Supplementary Figure 2.** The PSDs of the noised bipolar recordings before and after the ECG artifacts were removed.

# The estimation of the online template subtraction

We tested the windows for 30s, 20s, 10s, and 5s, respectively. The offline data was segmented equally to the window (for example, 30s). Then a template was extracted from a data segment and was subtracted. We calculated the averaged PSDs of the segments before and after removing. The performance is shown in Supplementary Figure 3. The results suggested that the signal-noise-ratio of the cleaned segments significantly decreased when the window is shorter than 20s. Power at 23Hz significantly attenuated in the cleaned 20s-segments and 10s-segments because some power was remained in the template and was subtracted together with the ECG spikes. Thus, the template stayed valid in the window of 30s. Considering the interval of the simulated ECG spikes was 0.75s, the results indicate that at least 40 spikes were necessary for extracting a valid template. If the heart rate changes, the widow should be adjusted.


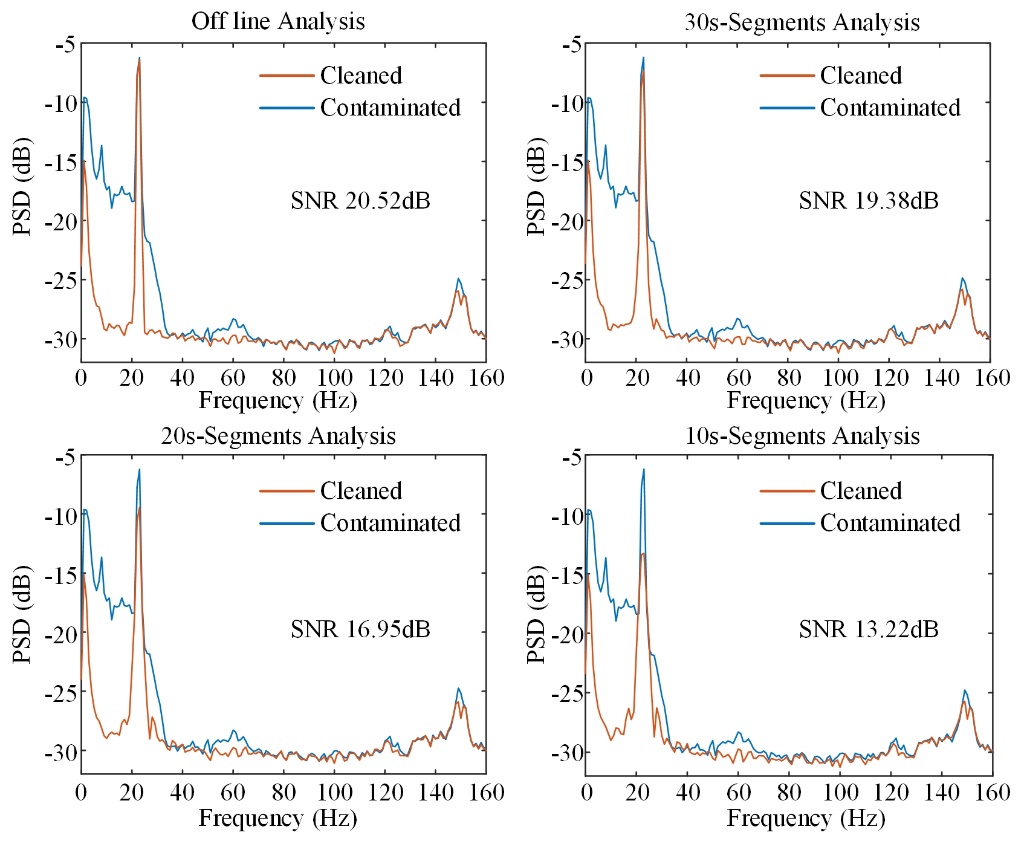


**Supplementary Figure 3.** The PSDs of the offline removal and online removal results. PSDs of the online results were averaged.
